# Pathogen transmission risk by opportunistic gulls moving across human landscapes

**DOI:** 10.1038/s41598-019-46326-1

**Published:** 2019-07-23

**Authors:** Joan Navarro, David Grémillet, Isabel Afán, Francisco Miranda, Willem Bouten, Manuela G. Forero, Jordi Figuerola

**Affiliations:** 1 Institut de Ciències del Mar - CSIC, Barcelona, Spain; 2 Centre d’Ecologie Fonctionnelle et Evolutive, UMR 5175, CNRS - Université de Montpellier, Université Paul-Valéry Montpellier - EPHE, Montpellier, France; 3 FitzPatrick Institute, University of Cape Town, Rondebosch, South Africa; 4 Estación Biológica de Doñana - CSIC, Sevilla, Spain; 5 Computational Geo-Ecology, Institute for Biodiversity and Ecosystem Dynamics (IBED), University of Amsterdam, Amsterdam, The Netherlands; 6 CIBER Epidemiología y Salud Pública (CIBER-ESP), Sevilla, Spain

**Keywords:** Ecology, Pathogens, Ecology, Behavioural ecology

## Abstract

Wildlife that exploit human-made habitats hosts and spreads bacterial pathogens. This shapes the epidemiology of infectious diseases and facilitates pathogen spill-over between wildlife and humans. This is a global problem, yet little is known about the dissemination potential of pathogen-infected animals. By combining molecular pathogen diagnosis with GPS tracking of pathogen-infected gulls, we show how this knowledge gap could be filled at regional scales. Specifically, we generated pathogen risk maps of *Salmonella*, *Campylobacter* and *Chlamydia* based on the spatial movements of pathogen-infected yellow-legged gulls (*Larus michahellis*) equipped with GPS recorders. Also, crossing this spatial information with habitat information, we identified critical habitats for the potential transmission of these bacteria in southern Europe. The use of human-made habitats by infected-gulls could potentially increase the potential risk of direct and indirect bidirectional transmission of pathogens between humans and wildlife. Our findings show that pathogen-infected wildlife equipped with GPS recorders can provide accurate information on the spatial spread risk for zoonotic bacteria. Integration of GPS-tracking with classical epidemiological approaches may help to improve zoonosis surveillance and control programs.

## Introduction

Wild animals host and spread pathogens, thereby shaping the epidemiology of infectious diseases^1–3^. This is particularly relevant in human-transformed landscapes, where opportunistic species reach high densities associated with the exploitation of anthropogenic food sources that could carry pathogenic bacteria^4–7^. This facilitates pathogen spill-over between wildlife and humans, both ways, and there are concerns that this may facilitate the evolution of new zoonotic pathogens^6,8–10^. Notably, urban gulls threaten public health because they shed bacterial pathogens, antibiotic-resistant bacteria, and viruses^5,11–13^. This has become a public health problem, yet little is known about how gulls spread zoonoses in space and time^5,13,14^. The lack of information on the dissemination process of zoonotic pathogens weakens risk assessments and management plans^15^. Specifically, spatially-explicit wildlife epidemiology is missing from existing zoonosis surveillance and control actions, such as the Zoonosis Directive of the European Union^16^ and the Foodborne Diseases Active Surveillance Network in the USA^17^.

We determined how this gap could be filled at a regional scale, by coupling conventional pathogen diagnosis in gulls with GPS-tracking of bird movements using miniature electronic tags attached to infected individuals. This allows the compilation of pathogen risk maps and the identification of critical habitats, as we show for yellow-legged gulls (*Larus michahellis*) in southern Spain. Due to its scavenger habits, this gull has been reported as a source and reservoir of zoonotic pathogens^18,19^. We GPS-tracked 14 birds that tested positive for one of three major zoonotic bacteria (five *Salmonella*-infected, five *Campylobacter*-infected and four *Chlamydia*-infected gulls). These bacteria are leading causes of zoonotic diseases in developed and developing countries^17,20^, and their incidence is increasing, even in countries with adequate public health systems. For example, *Salmonella* and *Campylobacter* cause the most common enteric zoonoses in the European Union, with 94,530 and 246,307 human clinical infections in 2016, respectively^20^. In the case of *Chlamydia*, this bacterium could affect the respiratory system of humans, wildlife and domestic animals^21^.

## Results and Discussion

**Figure 1:**
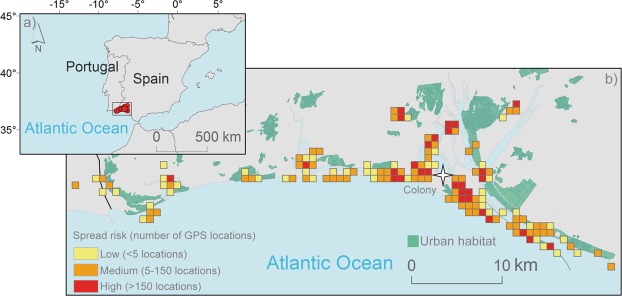
(**a**) Study area showing the terrestrial GPS positions (red circles) of 14 GPS-tracked yellow-legged gulls during the 2015 breeding season. (**b**) Potential risk maps for *Salmonella*, *Campylobacter*, and *Chlamydia* together, based on the spatial distribution of pathogen-infected yellow-legged gulls. The white star indicates the position of the breeding colony.

Cloacal swabs revealed that within the 19 GPS-tracked individuals, 37% (n = 5), 31% (n = 5) and 25% (n = 4) were positive for *Salmonella*, *Campylobacter* and *Chlamydia*, respectively, with no co-infections recorded. Previous studies found similar infection rates^18,22^. All movements of the infected-gulls were recorded throughout their estimated infection period [30 days^23–25^]. Pathogen risk maps and critical habitats were modeled by overlapping gull resting and foraging positions with accurate high-resolution land cover information^26,27^. The 27,798 recorded GPS locations revealed the greatest bacterial spread risk within 5 km of the breeding colony (Figs 1 and S1 in Supplementary Material), without significant differences in the type of habitat used between S*almonella*-infected, *Campylobacter*-infected and *Chlamydia*-infected individuals (Pseudo-*F* = 0.78, p = 0.67). Risk spatial extent varied between infected-gulls (Fig. S1 in Supplementary Material), from areas close to the breeding colony to some infected-gull crossing-over from Spain to Portugal, stressing the importance of international health regulations and cooperation in disease control^28^.

**Figure 2:**
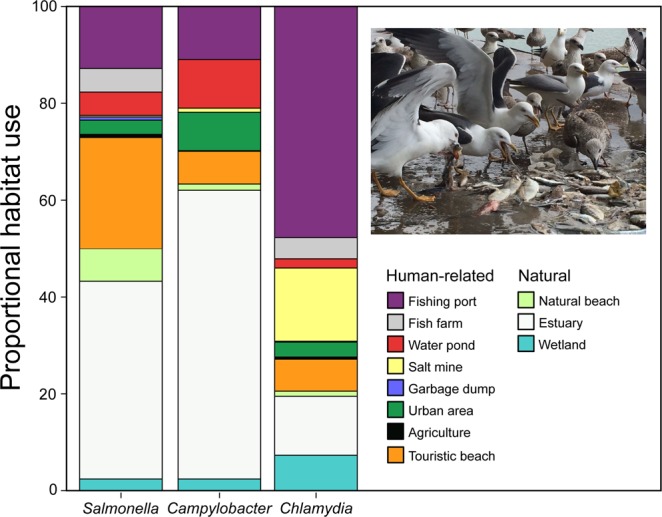
Average habitat use of *Salmonella*-infected, *Campylobacter*-infected and *Chlamydia*-infected yellow-legged gulls GPS-tracked during the 2015 breeding season. Each pathogen is represented by a vertical bar, subdivided by the proportion of locations in each habitat (human-related or natural) in relation to all GPS positions. The picture shows a group of adult and juvenile yellow-legged gulls feeding on fish refuse at a fishing port close to the breeding colony. Photograph taken by Joan Navarro in a fishing port close to the breeding colony.

Spread-risk areas overlapped with human-related habitats such as water ponds, fishing port or touristic beaches (Figs 2; S2 in Supplementary Material), increasing the risk of direct and indirect disease transmission to and from humans^10,14^ Notably, the use of water reservoirs (built for human use) by infected gulls is likely to lead to the contamination of drinking, recreational and irrigation water sources^29^. For this reason, it is important to ensure correct water treatment in this sensible habitats to reduce any potential risk to public health. Similarly, the extensive use of fishing ports and fish farms as feeding areas by yellow-legged gulls could point to serious infection risk for seafood^30^. Moreover, the use of beaches by infected-gulls (Fig. 2) exposes to pathogen spillover tens of thousands of tourists using these recreational habitats^14^. Moreover, the utilization of wetlands or estuaries by infected-gulls enhances the probability for pathogen transmission to other wildlife species^31^. Garbage dumps are also assumed to facilitate the infection of gulls by pathogens present in the human organic garbage, as well as cross-species and cross-individual transmission^13,18^. Yet, this habitat was seldom used by gulls in our study, due to its low availability in the area used by tracked-gulls (there are only two dumps in the area surrounding the breeding colony^27^). If garbage dumps are not the main pathogen source, bacterial infection of GPS-tracked gulls may be associated with the use of other food sources in decomposition, such as stranded marine animals (notably mammals) that could present pathogenic-bacteria, human organic refuse food found in recreational beaches or urban parks, or urban prey such as pigeons and rats^32,33^. Our results strongly indicate the need for integrated waste and pest control at a landscape scale.

Overall, our study reveals that pathogen-infected gulls equipped with GPS recorders could provide accurate maps of zoonotic spread risk, from the local to regional and international scales. In some circumstances, this approach could be scaled up to build an international network, using gulls and other potential vectors of animal pathogens^34^, to achieve large scale zoonotic surveillance and to identify and implement prevention measures across potential sensitive habitats. Because this may trigger public concern, we recommend that these measures be coupled with environmental mediation work, to ensure that wildlife is not perceived as generally harmful to humans^35^.

## Material and Methods

### Fieldwork and tracking procedures

Fieldwork was carried out at the natural Biosphere reserve of Marismas de Odiel (37°13′N, 6°59′W; southwestern Iberian Peninsula; Fig. 1) in a colony of 250–300 breeding pairs of yellow-legged gulls. We deployed high-resolution GPS-trackers recording the positions of individuals at 5 minute intervals [Uva-Bits loggers^36^] on 19 breeding gulls more than 4-years of age during their breeding period (May 2015). Uva-BiTS loggers can recharge themselves using solar energy, allowing to track the movements of birds continuously during several years^36^. The age of each individual was determined from plumage characteristics. Incubating birds were caught at the nest using a walk-in wire mesh trap and GPS-trackers were attached using a wing harness fixed with a reef knot in the tracheal pit, an attachment method recommended for large gulls^37^. The GPS-tracker and harness weighed less than 1.8% of the body mass of the birds [16 g for the GPS and harness, mean ± standard deviation = 1072 ± 110 g for the tracked gulls]^26^. GPS data were automatically downloaded remotely from devices to a field-based laptop when the birds were present at the breeding colony, where a network of 3 antennas provided complete coverage of the breeding area^36^. GPS data was parsed into the central database and immediately available in the UvA-BiTS Virtual Lab (www.UvA-BiTS.nl) for visualization and data exploration, therefore providing tracking information in real time^36^. To avoid potential biases associated with differences in the number of GPS data between individuals, tracking data were analyzed only when all individuals were equipped. We focused our analyses on the 30 days following deployment (from 14-May to 15-June 2015) to cover the potential infection period of each tracked pathogen^23–25^.

All fieldwork was approved by the Ethics Committee of CSIC (Ref: 28-04-15-237), in accordance with the Spanish and EU legislation on the protection of animals used for scientific purposes.

### Pathogen determination

Cloacal swabs from each GPS-tracked gull were collected and placed in PBS medium (Deltalab, Barcelona, Spain), and stored frozen at −80 °C. The detection of each pathogen was performed in the Ecophysiology Laboratory of the Estación Biológica de Doñana CSIC (http://ebd.csic.es/lef/web/) using real-time PCR assays for each bacterial genus (*Salmonella*, *Campylobacter* and *Chlamydia*) following established protocols^38–40^. Before each PCR assay, DNA was extracted from each cloacal swab using a commercial DNA purification kit (Promega Maxwell^®^). CT values of 40 were used as cut-off points. As we used non-specific PCR primers, we only detected the genus of the pathogen. We selected these three bacteria because they are leading causes of zoonotic enteric diseases (*Salmonella* and *Campylobacter*) and respiratory diseases (*Chlamydia*) in developed and developing countries, affecting humans, wildlife and domestic animals^20,21^. The primers for *Salmonella* were able to detect 99.4% of 630 strains belonging to over 100 serovars^40^. The primers for Campylobacter successfully amplify *C. jejuni* and *C. coli*, but not other Campylobacter species. The primers for *Chlamydia* and *Chlamydophila* successfully detect the nine known species for these genus. However, as we only evaluate the presence of these bacteria at genus level, we unknown if all individuals infected with *Salmonella*, *Campylobacter* or *Chlamydia* are really infected with pathogens that can also infect humans.

### Potential pathogen risk maps and habitat use

We only considered locations recorded outside the gull breeding colony (using a radius of 500 m around each nest. see^26^). Further, we assumed that gulls mainly shed pathogens to the environment through their feces. Consequently, a high risk of infection was assumed to occur within feeding and resting areas. Therefore, we removed all locations associated with gull travelling behavior [speed >4 km·h^−1^]^26^ and those location on the sea. Habitat use was assigned to each gull location by overlapping locations with land cover information. High resolution information on land cover was obtained from the program SIOSE (Soil Information System of Spain, Junta de Andalucía, last update 2013) and geographical references of waste dumps from the Spatial Reference Databases of Andalucía (DERA, last update 21/02/2014). This habitat classification was subsequently visually reviewed using the most recent satellite images offered by Google Earth V 7.1.2.2041 at a 0.5 m spatial resolution. All GPS foraging locations were finally classified into eleven categories: Estuary, wetland, touristic beach, natural beach, fishing port, salt mine, fish farm, water pond, agricultural area, urban area and garbage dump. Pathogen risk maps were constructed on the basis of the current distribution of GPS-tracked gulls infected by each pathogen. The transmission risk was estimated from the number of locations of infected gulls collected on a spatial grid of 750 × 750 m over the entire study area. Differences in habitat use (%) between *Salmonella*-infected, *Campylobacter*-infected and *Chlamydia*-infected yellow-legged gulls were tested using one way semiparametric permutation multivariate analyses of variance tests (PERMANOVA tests) on the Euclidean distance matrix^41^. PERMANOVA allows for the analysis of statistical designs without the constraints of multivariate normality, homoscedasticity and greater number of variables than sampling units. The method calculates a pseudo-F-statistic directly analogous to the traditional F-statistic for ANOVA tests, using permutation procedures to obtain P-values for each term in the model^41^.

## Supplementary information

Fig. S1 and Fig. S2

## Data Availability

All data are available in a central PostgreSQL database at UvA-BiTS (http://www.uva-bits.nl/virtual-lab).
