# Supplementary material for: Pathogen transmission risk by opportunistic gulls moving across human landscapes
Source: Sci Rep. 2019 Jul 23;9:10659. doi: 10.1038/s41598-019-46326-1 (PMC6650491; doi:10.1038/s41598-019-46326-1)

**Supplementary Information for:**

**Pathogen transmission risk by opportunistic gulls moving across human landscapes**

Joan Navarro^1,*^, David Grémillet^2,3^, Isabel Afán^4^, Francisco Miranda^4^, Willem Bouten^5^, Manuela G. Forero^4^ and Jordi Figuerola^4,6^

**Figure S1.** Aggregated summary of GPS locations of yellow-legged gulls (*Larus michahellis*) infected by a) *Salmonella*, b) *Campylobacter*, and c) *Chlamydia*. Yellow-legged gulls were GPS-tracked during the 2015 breeding season.


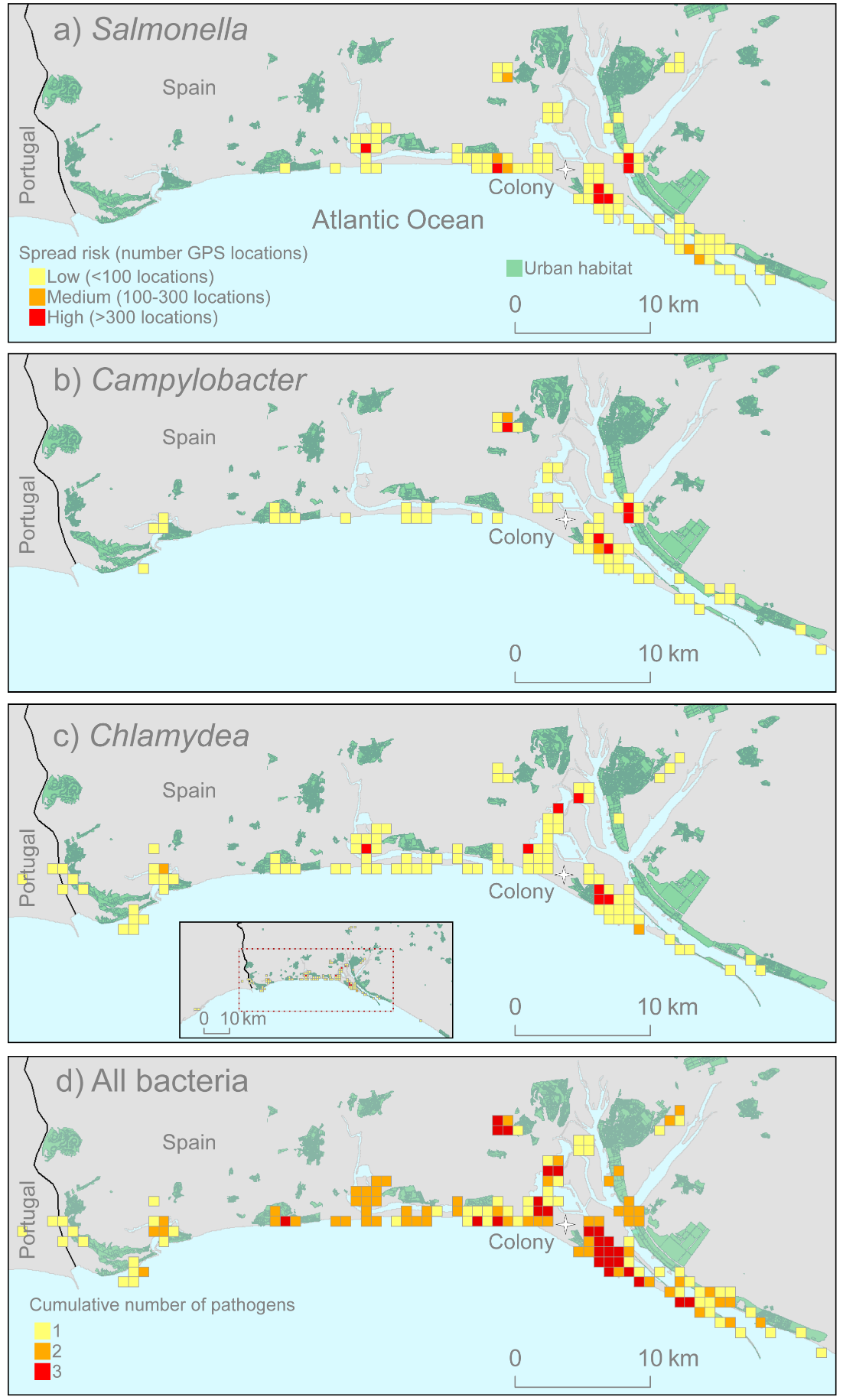


**Figure S2**. Spatial distribution of the habitats used by yellow-legged gulls equipped with GPS loggers while breeding at the natural protected Biosphere Reserve of Marismas de Odiel (southwestern Iberian Peninsula, Spain). Habitat information was drawn from the SIOSE database (Soil Information System of Spain, Junta de Andalucía, last update 2013). Zooms onto the different habitats used by yellow-legged gulls were also indicated. The map is made with ArcGIS 10.3 software (ESRI; academic licenses provided by CSIC). Images were freely provided by the Spanish National Geographic Institute: OrtoPNOA 2014 CC-BY 4.0 [scne.es](http://www.scne.es/); for information about open access based on Creative Commons License CC-By 4.0 see Spanish Government normative in <http://www.ign.es/resources/licencia/Condiciones_licenciaUso_IGN.pdf>).


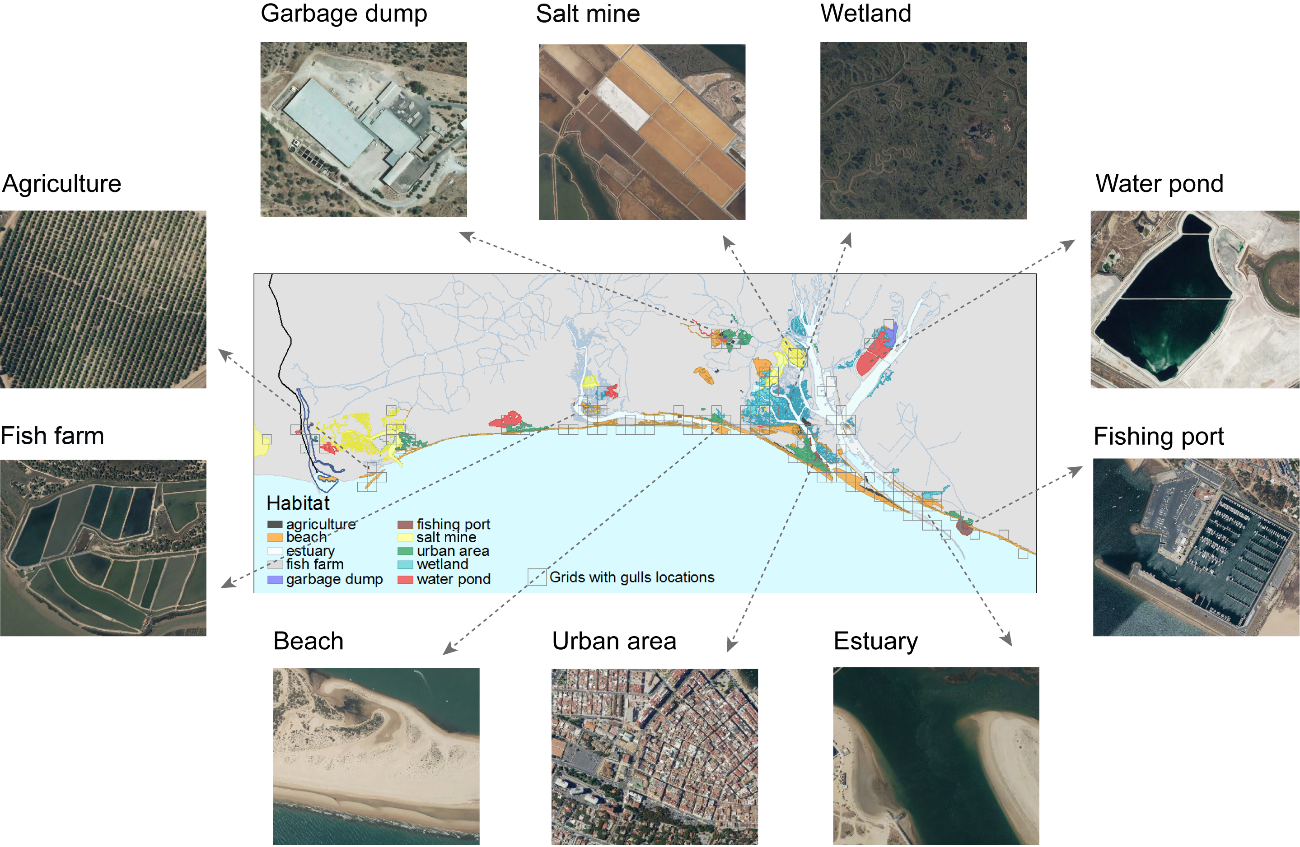

Supplement: Supplementary file 1 — Fig. S1 and Fig. S2 [file 41598_2019_46326_MOESM1_ESM.doc]
